# [4-(All­yloxy)phen­yl](phen­yl)methanone

**DOI:** 10.1107/S1600536814014342

**Published:** 2014-06-25

**Authors:** Richard F. D’Vries, Carlos D. Grande, Manuel N. Chaur, Javier A. Ellena, Rigoberto C. Advincula

**Affiliations:** a Instituto de Física de São Carlos, Universidade de São Paulo, Av. Trabalhador são-carlense 400, São Carlos, SP, 13566-590, Brazil; b Programa de Ingenieria Agroindustrial, Universidad San Buenaventura, AA 7154, Santiago de Cali, Colombia; c Departamento de Química, Facultad de Ciencias, Universidad del Valle, AA 25360, Santiago de Cali, Colombia; d Case Western Reserve University, Department of Macromolecular Science and Engineering, 2100 Adelbert Road, Kent Hale Smith Bldg, Cleveland, Ohio 44106, USA

**Keywords:** crystal structure

## Abstract

The structure of the title compound, C_16_H_14_O_2_, features a dihedral angle of 54.4 (3)° between the aromatic rings. The allyl group is rotated by 37.4 (4)° relative to the adjacent benzene ring. The crystal packing is characterized by numerous C—H⋯O and C—H⋯π inter­actions. Most of these inter­actions occur in layers along (011). The layers are linked by C—H⋯π inter­actions along [100], forming a three-dimensional network.

## Related literature   

For more details of the synthesis, see: Prucker *et al.* (1999 ▶). For photoreactive properties of benzo­phenone derivates, see: Shirahata & Kishimoto (1984 ▶); Dorman & Prestwich (1994 ▶); Beckett & Porter (1963 ▶); Kubo *et al.* (2010 ▶); Balakirev *et al.* (2005 ▶); Ferreira *et al.* (1995 ▶); Matsushita *et al.* (1992 ▶). For related structures, see: Schlemper (1982 ▶); Norment & Karle (1962 ▶); Guo *et al.* (1992 ▶).
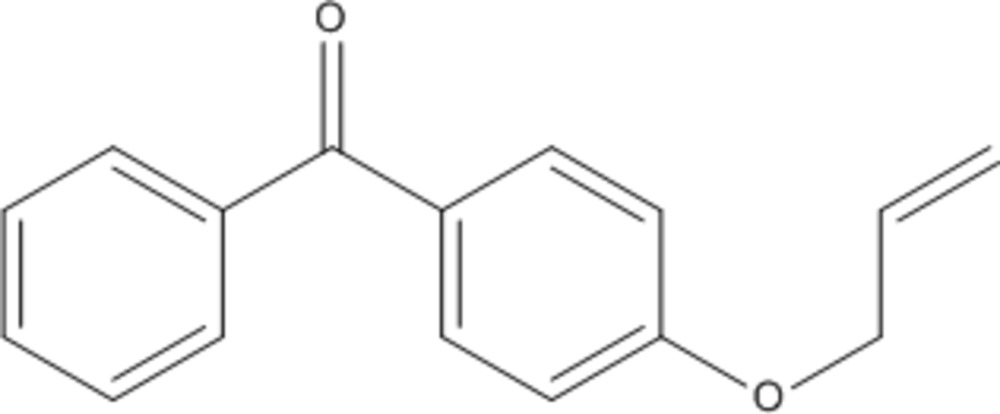


## Experimental   

### 

#### Crystal data  

C_16_H_14_O_2_
*M* *_r_* = 238.27
Monoclinic, 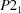

*a* = 6.0141 (5) Å
*b* = 7.8839 (8) Å
*c* = 13.5992 (14) Å
β = 94.442 (6)°
*V* = 642.86 (11) Å^3^
*Z* = 2
Mo *K*α radiation
μ = 0.08 mm^−1^
*T* = 293 K
0.12 × 0.08 × 0.06 mm

#### Data collection  

Nonius KappaCCD diffractometer
1609 measured reflections
1603 independent reflections
1021 reflections with *I* > 2σ(*I*)
*R*_int_ = 0.065

#### Refinement  

*R*[*F* ^2^ > 2σ(*F* ^2^)] = 0.049
*wR*(*F* ^2^) = 0.133
*S* = 1.01
1603 reflections
167 parameters
1 restraint
H atoms treated by a mixture of independent and constrained refinement
Δρ_max_ = 0.17 e Å^−3^
Δρ_min_ = −0.16 e Å^−3^

### 

Data collection: *COLLECT* (Nonius, 1998 ▶); cell refinement: *SCALEPACK* (Otwinowski & Minor, 1997 ▶); data reduction: *DENZO* (Otwinowski & Minor, 1997 ▶) and *SCALEPACK*; program(s) used to solve structure: *SHELXS2013* (Sheldrick, 2008 ▶); program(s) used to refine structure: *SHELXL2013* (Sheldrick, 2008 ▶); molecular graphics: *ORTEP-3 for Windows* (Farrugia, 2012 ▶); software used to prepare material for publication: *WinGX* (Farrugia, 2012 ▶), *DIAMOND* (Brandenburg, 2006 ▶), *Mercury* (Macrae *et al.*, 2008 ▶) and *PARST* (Nardelli, 1995 ▶).

## Supplementary Material

Crystal structure: contains datablock(s) global, I. DOI: 10.1107/S1600536814014342/ld2129sup1.cif

Structure factors: contains datablock(s) I. DOI: 10.1107/S1600536814014342/ld2129Isup2.hkl

Supporting information file. DOI: 10.1107/S1600536814014342/ld2129Isup3.cml

CCDC reference: 1009057

Additional supporting information:  crystallographic information; 3D view; checkCIF report

## Figures and Tables

**Table 1:** Hydrogen-bond geometry (Å, °) *Cg*1 and *Cg*2 are the centroids of the C1–C6 and C8–C13 rings, respectively.

*D*—H⋯*A*	*D*—H	H⋯*A*	*D*⋯*A*	*D*—H⋯*A*
C5—H5⋯*Cg*1^i^	0.93	2.83	3.651 (3)	147
C14—H14*B*⋯*Cg*2^ii^	0.97	2.96	3.630 (3)	127
C10—H10⋯O2^ii^	0.93	2.89	3.528 (4)	127
C2—H2⋯O1^iii^	0.93	2.85	3.698 (4)	152
C14—H14*B*⋯O1^iv^	0.97	2.85	3.596 (4)	134

Symmetry codes: (i) 
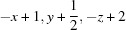
; (ii) 
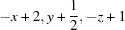
; (iii) 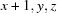; (iv) 
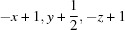
.

**Fig. 1.**
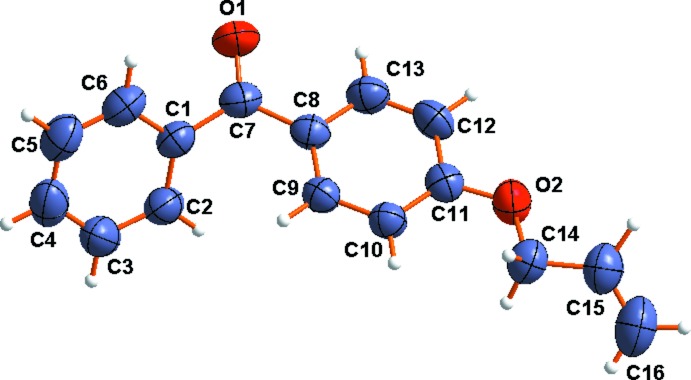
The ORTEP structure of the title compound with displacement ellipsoid plot drawn at the 50% probability level.

**Fig. 2.**
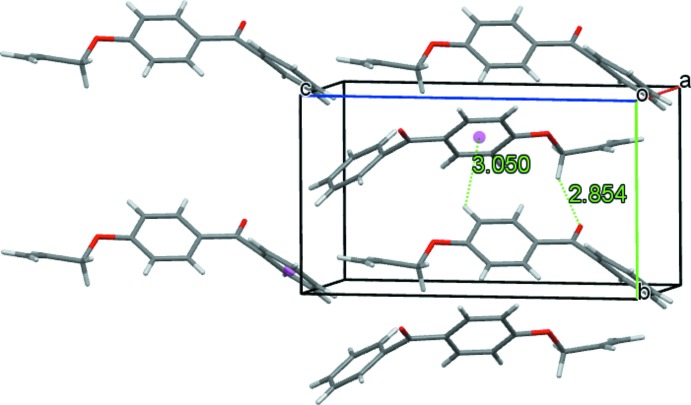
Crystal packing view of the title compound along [100] direction. The hydrogen bonds are shown as dashed lines.

**Fig. 3.**
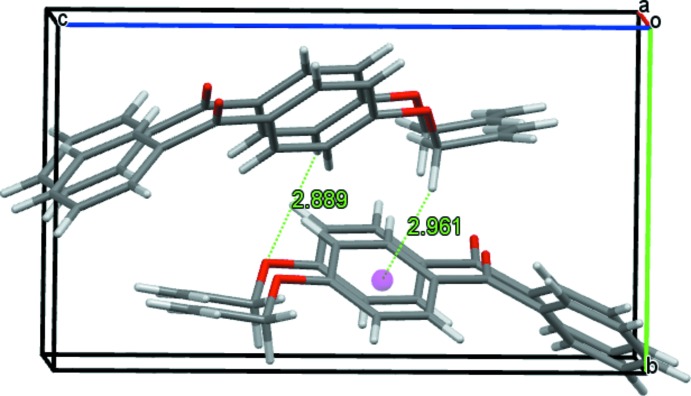
Crystal packing view of the title compound along [001] direction. The hydrogen bonds are shown as dashed lines.

## supplementary crystallographic information

### S1. Introduction

The title compound C_16_H_14_O_2_, is based on a photoreactive benzo­phenone derivative that can be bound to SiO_2_ surfaces *via* a silane anchor. This substrate is a compound that has a benzo­phenone moiety which exhibits a known photoreactivity and is particularly useful in photopolymerizable organopolysiloxane and silicone resins Prucker *et al.*, (1999); Shirahata & Kishimoto, (1984); Dorman & Prestwich, (1994); Beckett & Porter, (1963). When triggered by UV light (λ = 365 nm), a biradical triplet state is formed, which is able of abstracting a proton from any neighboring aliphatic C—H group to form a C—C bond (Ferreira *et al.*, (1995); Balakirev *et al.*, (2005); Kubo *et al.* (2010)) and as a result of the photochemical reaction, a thin layer of the polymer is covalently bound to the surface (Prucker *et al.* (1999); Shirahata & Kishimoto, (1984); Dorman & Prestwich, (1994); Matsushita *et al.* (1992)). This molecule can be also copolymerized with other polymer molecules and will not migrate out through its double bond.

### S2. Experimental

Synthesis of (4-Allyl­oxy-phenyl)-phenyl-methanone. This compound was synthesized by a procedure already reported. A mixture of 4-hy­droxy­benzo­phenone (39.6 g, 0.2 mol) and allyl bromide (26.6 g, 0.22 mol) were dissolved in 120 mL of acetone and 28 g of potassium carbonate. The mixture was heated to reflux for 8 h and then cooled down to room temperature. Water (80 mL) was added and the resulting solution was extracted twice with 100 mL of di­ethyl ether. The combined organic phases were washed with 100 mL of aqueous NaOH (10%) and dried over Na_2_SO_4_, and the solvent was evaporated. The resulting yellowish product was purified from methanol to yield 40 g (89%) of pure product as confirmed by NMR. FTIR (KBr): 3081, 3059, 3022, 2939, 2865, 1650, 1600 cm^-1^. ^1^H NMR (CDCl_3_, δ(ppm): 4.6 (m, 2H, OCH_2_), 5.3-5.5 (m, 2H, CH_2_=), 6.1 (m, 1H,=CH-), 7.21 (2H, m); 7.44 (2H, m); 7.56 (1H, m); 7.77 (2H, m); 7.88 (2H, m). ^13^C NMR: δ(ppm) in CDCl_3_: 195.13 (C=O), 162.14 (OCarom), 149.12, 137.60, 135.94, 132.78, 131.93, 130.19, 128.35, 118.29, 115.91, 69.34 (OCH_2_).

### S2.1. Refinement

All H atoms were placed in idealized positions, with C—H bond lengths fixed to 0.93 (aromatic C—H) or 0.97 Å (terminal methyl­ene), and refined as riding with displacement parameters calculated as Uiso(H) = xUeq(carrier C) where x = 1.2.

### S3. Results and discussion

The compound C_16_H_14_O_2_ is a benzo­phenone derivate formed by two aromatic rings linked by a ketone funtion. The presence of the ketone funtion and an allyl­oxy group make of this molecule particularly useful in photopolymerizable organopolysiloxane and silicone resins, and a excellent substrate for selective catalysis. The rings are twisted with a torsion angle formed between C6—C1—C7—C8 of 136.59 (31)°. The allyl­oxy group presents a torsion angle of -7.99 (42)° (C10—C11—O2—C14) relative to the aromatic ring. The molecules inter­act *via* supra­molecular weak inter­actions C5—H5···π = 3.651 (3) , C14—H14B···O1 = 3.596 (4) and C10—H10··O2 = 3.528 (4) Å giving rise to supra­molecular layers in the plane (011). C14—H14B···π = 3.698 (2) Å inter­action join the layers along [100] to obtain the supra­molecular crystal packing.

### Crystal data

**Table:**

C_16_H_14_O_2_	*F*(000) = 252
*M**_r_* = 238.27	*D*_x_ = 1.231 Mg m^−^^3^
Monoclinic, *P*2_1_	Mo *K*α radiation, λ = 0.71073 Å
Hall symbol: P 2yb	Cell parameters from 1583 reflections
*a* = 6.0141 (5) Å	θ = 3.0–27.9°
*b* = 7.8839 (8) Å	µ = 0.08 mm^−^^1^
*c* = 13.5992 (14) Å	*T* = 293 K
β = 94.442 (6)°	Prism, colourless
*V* = 642.86 (11) Å^3^	0.12 × 0.08 × 0.06 mm
*Z* = 2	

### Data collection

**Table:**

Nonius KappaCCD diffractometer	1021 reflections with *I* > 2σ(*I*)
Radiation source: Enraf–Nonius FR590	*R*_int_ = 0.065
Horizonally mounted graphite crystal monochromator	θ_max_ = 27.8°, θ_min_ = 3.0°
Detector resolution: 9 pixels mm^-1^	*h* = 0.000000→7.000000
CCD rotation images, thick slices scans	*k* = 0.00000→10.000000
1609 measured reflections	*l* = −17.00000→17.000000
1603 independent reflections	

### Refinement

**Table:**

Refinement on *F*^2^	0 constraints
Least-squares matrix: full	Hydrogen site location: inferred from neighbouring sites
*R*[*F*^2^ > 2σ(*F*^2^)] = 0.049	H atoms treated by a mixture of independent and constrained refinement
*wR*(*F*^2^) = 0.133	*w* = 1/[σ^2^(*F*_o_^2^) + (0.0858*P*)^2^] where *P* = (*F*_o_^2^ + 2*F*_c_^2^)/3
*S* = 1.01	(Δ/σ)_max_ < 0.001
1603 reflections	Δρ_max_ = 0.17 e Å^−^^3^
167 parameters	Δρ_min_ = −0.16 e Å^−^^3^
1 restraint	

### Special details

**Table:**

Experimental. The absence of some reflections of the data sets is due to merged them. The 001 reflection was removed because it intensity was affect by the beam stop.
Geometry. All e.s.d.'s (except the e.s.d. in the dihedral angle between two l.s. planes) are estimated using the full covariance matrix. The cell e.s.d.'s are taken into account individually in the estimation of e.s.d.'s in distances, angles and torsion angles; correlations between e.s.d.'s in cell parameters are only used when they are defined by crystal symmetry. An approximate (isotropic) treatment of cell e.s.d.'s is used for estimating e.s.d.'s involving l.s. planes.

### Fractional atomic coordinates and isotropic or equivalent isotropic displacement parameters (Å^2^)

**Table:**

	*x*	*y*	*z*	*U*_iso_*/*U*_eq_	
C16	1.2957 (8)	0.3081 (6)	0.2215 (3)	0.1064 (13)	
H16A	1.4203	0.317	0.2662	0.128*	
H16B	1.3132	0.2899	0.155	0.128*	
H15	0.960 (6)	0.322 (7)	0.199 (3)	0.119 (14)*	
C1	0.5814 (4)	0.3364 (4)	0.8323 (2)	0.0589 (7)	
C2	0.7988 (4)	0.3074 (4)	0.8703 (2)	0.0660 (8)	
H2	0.8946	0.2419	0.8356	0.079*	
C3	0.8714 (5)	0.3768 (5)	0.9602 (2)	0.0780 (9)	
H3	1.0159	0.3552	0.9867	0.094*	
C4	0.7337 (6)	0.4775 (5)	1.0114 (2)	0.0846 (10)	
H4	0.7851	0.5244	1.0717	0.102*	
C5	0.5206 (6)	0.5084 (5)	0.9730 (3)	0.0825 (9)	
H5	0.4277	0.5779	1.0068	0.099*	
C6	0.4430 (5)	0.4374 (5)	0.8849 (2)	0.0732 (8)	
H6	0.2967	0.457	0.8601	0.088*	
C7	0.4818 (4)	0.2537 (4)	0.7404 (2)	0.0618 (7)	
C8	0.6023 (4)	0.2502 (3)	0.64903 (19)	0.0551 (6)	
C9	0.7921 (4)	0.3450 (4)	0.63562 (19)	0.0558 (6)	
H9	0.8546	0.4107	0.6874	0.067*	
C10	0.8906 (4)	0.3438 (3)	0.54670 (18)	0.0577 (6)	
H10	1.0187	0.4071	0.5394	0.069*	
C11	0.7969 (4)	0.2475 (4)	0.46872 (19)	0.0570 (6)	
C12	0.6079 (4)	0.1514 (4)	0.4819 (2)	0.0666 (8)	
H12	0.5458	0.0852	0.4302	0.08*	
C13	0.5125 (4)	0.1529 (4)	0.5696 (2)	0.0656 (7)	
H13	0.3855	0.0881	0.5767	0.079*	
C14	1.0531 (5)	0.3498 (4)	0.3553 (2)	0.0700 (8)	
H14A	1.1858	0.3269	0.3984	0.084*	
H14B	1.0095	0.4668	0.3647	0.084*	
C15	1.0986 (7)	0.3210 (6)	0.2518 (2)	0.0848 (10)	
O1	0.2952 (3)	0.1918 (4)	0.74038 (17)	0.0906 (8)	
O2	0.8762 (3)	0.2378 (3)	0.37801 (13)	0.0715 (6)	

### Atomic displacement parameters (Å^2^)

**Table:**

	*U*^11^	*U*^22^	*U*^33^	*U*^12^	*U*^13^	*U*^23^
C16	0.136 (3)	0.105 (3)	0.082 (2)	0.012 (3)	0.032 (2)	−0.009 (2)
C1	0.0556 (14)	0.0588 (16)	0.0637 (14)	−0.0029 (13)	0.0135 (12)	0.0036 (14)
C2	0.0571 (15)	0.0756 (19)	0.0663 (17)	0.0002 (13)	0.0116 (13)	0.0027 (15)
C3	0.0674 (16)	0.103 (3)	0.0636 (17)	−0.0109 (17)	0.0026 (14)	0.0057 (17)
C4	0.098 (2)	0.093 (2)	0.0655 (18)	−0.0230 (19)	0.0192 (18)	−0.0085 (18)
C5	0.089 (2)	0.077 (2)	0.084 (2)	0.0005 (18)	0.0283 (18)	−0.0107 (18)
C6	0.0629 (15)	0.0760 (19)	0.083 (2)	0.0060 (15)	0.0220 (14)	0.0025 (18)
C7	0.0487 (14)	0.0666 (16)	0.0700 (16)	0.0002 (13)	0.0041 (12)	0.0027 (14)
C8	0.0513 (13)	0.0511 (14)	0.0624 (15)	0.0022 (12)	0.0006 (11)	0.0025 (13)
C9	0.0535 (13)	0.0560 (14)	0.0570 (14)	−0.0015 (12)	−0.0011 (11)	−0.0035 (13)
C10	0.0563 (13)	0.0584 (15)	0.0584 (15)	−0.0056 (13)	0.0038 (12)	0.0000 (14)
C11	0.0617 (14)	0.0521 (13)	0.0568 (15)	0.0042 (13)	0.0017 (12)	0.0011 (14)
C12	0.0700 (16)	0.0608 (16)	0.0671 (18)	−0.0082 (14)	−0.0076 (14)	−0.0089 (15)
C13	0.0603 (15)	0.0614 (16)	0.0751 (19)	−0.0101 (14)	0.0055 (14)	−0.0019 (16)
C14	0.0773 (18)	0.0667 (18)	0.0672 (17)	0.0032 (15)	0.0133 (14)	−0.0003 (16)
C15	0.099 (2)	0.092 (2)	0.0645 (17)	0.001 (2)	0.0139 (18)	0.0056 (18)
O1	0.0605 (12)	0.121 (2)	0.0911 (15)	−0.0256 (13)	0.0138 (11)	−0.0100 (15)
O2	0.0857 (13)	0.0694 (12)	0.0600 (11)	−0.0070 (11)	0.0099 (9)	−0.0039 (11)

### Geometric parameters (Å, º)

**Table:**

C16—C15	1.289 (5)	C8—C9	1.388 (4)
C16—H16A	0.93	C8—C13	1.399 (4)
C16—H16B	0.93	C9—C10	1.387 (4)
C1—C2	1.387 (3)	C9—H9	0.93
C1—C6	1.390 (4)	C10—C11	1.387 (4)
C1—C7	1.494 (4)	C10—H10	0.93
C2—C3	1.379 (4)	C11—O2	1.359 (3)
C2—H2	0.93	C11—C12	1.389 (4)
C3—C4	1.375 (5)	C12—C13	1.363 (4)
C3—H3	0.93	C12—H12	0.93
C4—C5	1.368 (5)	C13—H13	0.93
C4—H4	0.93	C14—O2	1.435 (3)
C5—C6	1.371 (5)	C14—C15	1.472 (4)
C5—H5	0.93	C14—H14A	0.97
C6—H6	0.93	C14—H14B	0.97
C7—O1	1.224 (3)	C15—H15	1.06 (4)
C7—C8	1.486 (4)		
			
C15—C16—H16A	120	C13—C8—C7	118.1 (2)
C15—C16—H16B	120	C8—C9—C10	121.5 (2)
H16A—C16—H16B	120	C8—C9—H9	119.3
C2—C1—C6	119.2 (3)	C10—C9—H9	119.3
C2—C1—C7	123.1 (3)	C11—C10—C9	119.6 (2)
C6—C1—C7	117.6 (2)	C11—C10—H10	120.2
C3—C2—C1	119.3 (3)	C9—C10—H10	120.2
C3—C2—H2	120.3	O2—C11—C10	125.1 (2)
C1—C2—H2	120.3	O2—C11—C12	115.7 (2)
C4—C3—C2	121.1 (3)	C10—C11—C12	119.2 (2)
C4—C3—H3	119.5	C13—C12—C11	120.8 (3)
C2—C3—H3	119.5	C13—C12—H12	119.6
C5—C4—C3	119.5 (3)	C11—C12—H12	119.6
C5—C4—H4	120.3	C12—C13—C8	121.1 (2)
C3—C4—H4	120.3	C12—C13—H13	119.4
C6—C5—C4	120.5 (3)	C8—C13—H13	119.4
C6—C5—H5	119.8	O2—C14—C15	107.8 (3)
C4—C5—H5	119.8	O2—C14—H14A	110.1
C5—C6—C1	120.4 (3)	C15—C14—H14A	110.1
C5—C6—H6	119.8	O2—C14—H14B	110.1
C1—C6—H6	119.8	C15—C14—H14B	110.1
O1—C7—C8	120.0 (3)	H14A—C14—H14B	108.5
O1—C7—C1	118.8 (3)	C16—C15—C14	124.2 (4)
C8—C7—C1	121.2 (2)	C16—C15—H15	119 (2)
C9—C8—C13	117.8 (2)	C14—C15—H15	117 (2)
C9—C8—C7	124.0 (2)	C11—O2—C14	118.6 (2)

### Hydrogen-bond geometry (Å, º)

Cg1 and Cg2 are the centroids of the C1–C6 and C8–C13 rings, respectively.

**Table:**

*D*—H···*A*	*D*—H	H···*A*	*D*···*A*	*D*—H···*A*
C5—H5···*Cg*1^i^	0.93	2.83	3.651 (3)	147
C14—H14*B*···*Cg*2^ii^	0.97	2.96	3.630 (3)	127
C10—H10···O2^ii^	0.93	2.89	3.528 (4)	127
C2—H2···O1^iii^	0.93	2.85	3.698 (4)	152
C14—H14*B*···O1^iv^	0.97	2.85	3.596 (4)	134

Symmetry codes: (i) −*x*+1, *y*+1/2, −*z*+2; (ii) −*x*+2, *y*+1/2, −*z*+1; (iii) *x*+1, *y*, *z*; (iv) −*x*+1, *y*+1/2, −*z*+1.
